# Doxycycline treatment for drug-resistant *Mycoplasma pneumoniae* pneumonia and evaluation of key predictive factors for refractory disease

**DOI:** 10.3389/fmed.2026.1714717

**Published:** 2026-04-08

**Authors:** Ruijuan Ren, Yuping Xu, Xinyan Cui, Mengsheng Zhang, Xiangtao Wu, Tuanjie Wang, Xue Liu, Shujun Li

**Affiliations:** Children’s Intensive Care Unit of the First Affiliated Hospital of Xinxiang Medical College, Xinxiang, Henan, China

**Keywords:** diagram, doxycycline, mycoplasma, refractory pneumonia, resistant

## Abstract

**Objective:**

Mycoplasma pneumoniae pneumonia (MPP) in children is a common respiratory infection, with rising concerns over macrolide-resistant strains. The aim of this study is to establish an effective diagnostic prediction model for refractory MPP (RMPP) and evaluate doxycycline as an alternative treatment for macrolide-unresponsive MPP (MUMPP).

**Methods:**

106 children with RMPP receiving doxycycline and 73 children with MUMPP treated with azithromycin were retrospectively analyzed. For all patients, the detection of MP-DNA in BALF, alongside clinical criteria, served to confirm active MP infection and infer phenotypic macrolide unresponsiveness/resistance. Univariate and multivariate logistic regression analyses were then used to identify clinically available risk factors for the development of RMPP among these patients with MP pneumonia.

**Results:**

The doxycycline group had significantly shorter fever duration, fewer lavage procedures, reduced steroid use, and shorter hospital stays compared to the azithromycin group (*P* < 0.05). Multivariate analysis identified four independent risk factors for RMPP: duration of macrolide use before admission, days of fever before hospitalization, procalcitonin (PCT), and C-reactive protein (CRP). A nomogram based on these factors showed excellent discrimination, with an AUC of 0.982 (95% CI: 0.961–1.000). The calibration curve approached the 45-degree line, and decision curve analysis (DCA) indicated that the nomogram provided positive net benefit across a reasonable range of threshold probabilities, supporting its potential clinical utility.

**Conclusion:**

In children with MUMPP, doxycycline treatment is associated with superior clinical outcomes compared to azithromycin. A nomogram incorporating readily available clinical factors can effectively identify patients at risk of developing RMPP, supporting early intervention.

## 1 Introduction

*Mycoplasma pneumoniae* pneumonia (MPP) represents a prevalent inflammatory pulmonary disease across pediatric age groups, caused by infection with *Mycoplasma pneumoniae* (MP). This condition constitutes approximately one-third of hospitalized cases of community-acquired pneumonia in pediatric patients (1). While the majority of pediatric patients with MPP exhibit mild symptoms that ameliorate within several days under standard therapy, a subset manifests with persistent fever, aggravated clinical manifestations, and deteriorating pulmonary radiographic findings, or even develops extrapulmonary complications after 7 days of standard therapy with macrolide antibiotics, evolving into what is termed refractory *Mycoplasma pneumoniae* pneumonia (RMPP). This condition exhibits an extended duration of pyrexia and hospitalization relative to conventional MPP, alongside an increased susceptibility to extrapulmonary complications, which can be severe and even life-threatening (2–4). Macrolides have long been recommended as the first-line antimicrobial agents for MPP management. Nonetheless, several studies indicate that the widespread use of macrolides has led to a rising prevalence of MUMPP, with resistance rates in Asia ranging approximately from 23.3 to 84.8% (5), and significant rates reported in other regions, making it a common clinical challenge. Consequently, clinicians frequently face the decision to escalate to second-line therapy. In the absence of appropriate treatment, MUMPP may progress to RMPP, thereby exacerbating the complexity of MUMPP management. Second-line antibiotics, such as tetracyclines (doxycycline or minocycline) and fluoroquinolones, have been recommended for the treatment of MUMPP (1). Although the availability of laboratory tests for macrolide resistance (e.g., PCR detection of 23S rRNA gene mutations), obtaining these results can take several days, creating a critical window where clinical decisions must be made based on evolving symptoms and risk assessment before definitive resistance data is available. Furthermore, access to such specialized testing is not universal across clinical settings. This underscores the clinical challenge of determining the optimal timing to initiate second-line antibiotics like doxycycline in a patient not responding to first-line macrolides, balancing the risks of delayed effective therapy against the unnecessary use of broader-spectrum agents. Despite the preliminary validation of doxycycline’s potential in treating MUMPP and RMPP, determining when to initiate doxycycline treatment in this context remains a significant clinical challenge. Among second-line options, doxycycline is often favored over fluoroquinolones in pediatric populations due to a more established safety profile for short-term use in children over 8 years of age, particularly regarding concerns about cartilage toxicity associated with fluoroquinolones. Compared to other tetracyclines, doxycycline is frequently preferred due to its better tolerability, twice-daily dosing, and lower propensity for phototoxicity than minocycline, contributing to its common use as a primary second-line agent for RMPP where tetracyclines are deemed appropriate.

In this study, we embarked on a retrospective analysis of the clinical symptoms, major laboratory tests, and radiological data of pediatric patients diagnosed with RMPP, aiming to determine the timing for initiating second-line antibiotic (doxycycline) treatment for pediatric MP pneumonia in the absence of macrolide resistance information. This analysis seeks to facilitate early identification of RMPP and furnish references for the selection of therapeutic regimens.

## 2 Materials and methods

### 2.1 General data

This study constitutes a retrospective analysis. The primary clinical comparison cohort consisted of 106 pediatric patients diagnosed with refractory Mycoplasma pneumoniae pneumonia (RMPP) who were admitted for doxycycline treatment (BALF) at the Department of Pediatrics of the First Affiliated Hospital of Xinxiang Medical University from September 2023 to December 2024 as the study group. Additionally, a cohort of 73 pediatric patients, hospitalized in the department of pediatrics of the same hospital and treated with azithromycin for MUMPP between April 2021 and December 2022, was selected as the control group. Therefore, a total of 179 patients (106 RMPP + 73 MUMPP) were included for the analysis of clinical outcomes and baseline characteristics (e.g., Table 1). For the specific purpose of developing the predictive model for RMPP risk, data from a focused subset of 44 pediatric patients from the RMPP group were utilized in the Lasso regression and subsequent model-building steps. All pediatric patients underwent bronchoalveolar lavage fluid (BALF) therapy upon admission, followed by quantitative detection of MP-DNA. The screening results, based on MP-DNA detection in BALF and the clinical definitions of treatment failure, indicated phenotypic macrolide resistance for patients in both groups. In the Meanwhile, the screening results revealed MP resistance, which was defined by a combination of clinical unresponsiveness to macrolide therapy (as per MUMPP/RMPP definitions) and confirmed by quantitative PCR (qPCR) detection of MP-DNA in BALF. While specific genetic testing for macrolide resistance mutations (e.g., in the 23S rRNA gene) was not routinely performed for all patients, the persistence of high MP-DNA load in BALF after standard macrolide treatment, along with the clinical criteria for MUMPP/RMPP, was used to infer phenotypic resistance.

**TABLE 1:** Comparison of baseline characteristics, clinical signs, and laboratory findings between the azithromycin-treated and doxycycline-treated pediatric patients with macrolide-unresponsive Mycoplasma pneumoniae pneumonia.

Variable (units)	Azithromycin group (*n* = 73)	Doxycycline group (*n* = 106)	U-value	*P*-value
Demographics				
Age (years)	8.42 (8.10, 8.92)	8.60 (8.10, 10.00)	1,776	0.3381
Clinical features at admission				
Peak temperature (°C)	39.50 (39.00, 40.00)	39.50 (39.20, 40.00)	1528.5	0.6631
Days of fever before hospital admission (days)	10.00 (7.00, 13.00)	10.00 (8.00, 13.25)	1,731	0.4823
Duration of macrolide antibiotic use outside hospital (days)	7.00 (5.00, 10.00)	8.00 (5.00, 11.00)	1,720	0.5213
Treatment outcomes				
Days of fever after hospital admission antibiotics (days)	**4.00 (2.00, 7.00)**	**3.00 (1.00, 5.25)**	**1,254**	**0.0468**
Number of lavages	**2.00 (1.00, 3.00)**	**1.00 (1.00, 2.00)**	**1,231**	**0.0222**
Length of hospital stay (days)	**12.00 (9.00, 17.00)**	**10.00 (7.00, 13.25)**	**1247.5**	**0.0433**
Duration of steroid use after hospital admission (days)	**7.00 (5.00, 10.00)**	**6.00 (5.00, 7.25)**	**1,249**	**0.0434**
Laboratory parameters				
Inflammatory markers				
CRP (mg/L)	20.65 (8.80, 45.00)	27.12 (8.05, 54.50)	1693.5	0.6244
PCT (ng/mL)	0.44 (0.22, 1.00)	0.74 (0.28, 1.77)	1856.5	0.1595
IL-6 (pg/mL)	21.10 (9.16, 44.85)	18.95 (12.69, 40.34)	1599.5	0.9731
Serum amyloid protein A (mg/L)	213.00 (55.40, 346.00)	250.50 (39.40, 508.25)	1,780	0.3289
Liver function and enzymes				
AST (U/L)	32.00 (26.00, 51.00)	28.00 (21.75, 40.25)	1313.5	0.1002
ALT (U/L)	20.00 (14.00, 44.00)	17.00 (13.00, 26.50)	1378.5	0.2011
LDH (U/L)	391.00 (258.00, 542.00)	292.50 (223.50, 439.50)	1273.5	0.0617
Creatine kinase (U/L)	48.00 (34.00, 106.00)	64.50 (37.00, 123.00)	1764.5	0.3739
Coagulation and other				
Fibrin degradation products (μg/mL)	5.90 (3.20, 16.10)	4.22 (2.35, 10.06)	1,336	0.1294
D-dimer (μg/mL)	2.90 (1.30, 6.00)	1.77 (0.80, 4.75)	1,316	0.1032
Hematology				
WBC (10^9^/L)	8.84 (6.90, 12.10)	8.55 (6.84, 10.60)	1,471	0.4491
Hemoglobin (g/L)	117.00 (112.00, 123.00)	120.00 (114.00, 123.75)	1,823	0.2227
Platelet count (10^9^/L)	333.00 (254.00, 394.00)	315.50 (236.50, 357.50)	1,375	0.1946
Neutrophil percentage (%)	69.60 (61.30, 79.00)	73.15 (63.48, 80.45)	1765.5	0.3709
Lymphocyte percentage (%)	22.80 (16.30, 32.00)	20.70 (15.43, 29.77)	1,435	0.3373
Mycoplasma load				
Mycoplasma load in pharyngeal swab (copies/mL)	1790.00 (500.00, 35500.00)	1051.00 (500.00, 24300.00)	1,619	0.9417
Mycoplasma load in BALF (copies/mL)	21500000.00 (781000.00, 130000000.00)	17350000.00 (3012500.00, 360500000.00)	1,786	0.3125

Data are presented as median (first quartile, third quartile). The Mann-Whitney U test was used for intergroup comparisons. Statistically significant differences (*P* < 0.05) are highlighted in bold. U-Value: Mann-Whitney U test statistic. Laboratory parameters were measured from blood samples collected within 24 h of admission, representing baseline status. CRP, C-reactive protein; PCT, procalcitonin; IL-6, interleukin-6; AST, aspartate aminotransferase; ALT, alanine aminotransferase; LDH, lactate dehydrogenase; WBC, white blood cell count; BALF, bronchoalveolar lavage fluid.

According to the suggestions in the “Guidelines for Diagnosis and Treatment of *Mycoplasma Pneumoniae* Pneumonia in Children (2023 Edition)” (1), the inclusion criteria in this study were as follows: (1) Manifestation of symptoms in all pediatric patients including cough and fever upon admission, coupled with the presence of novel infiltrates in chest X-rays; (2) Evidence of MP infection: positive serum-specific IgM antibodies and positive MP-DNA in BALF determined by fluorescent quantitative PCR (FQ-PCR); (3) Absence of infection evidence from other pathogens: negative bacterial cultures in sputum, BALF, and blood samples, and negative detection of respiratory viruses (respiratory syncytial virus, adenovirus, influenza virus, parainfluenza virus, metapneumovirus) in sputum; (4) Age of the pediatric patients being ≥ 8 years. The exclusion criteria were as follows: (1) Pediatric patients with a history of severe diseases, such as cranial trauma, epilepsy, hematologic diseases, autoimmune diseases, chromosomal disorders, metabolic diseases, end-stage renal disease, or chronic liver disease; (2) Pediatric patients with severe diseases or infections in other systems. RMPP diagnosis was defined as persistent fever, aggravated clinical manifestations, deteriorating pulmonary radiographic findings, and extrapulmonary complications after 7 days of standard therapy with macrolide antibiotics; MUMPP diagnosis was defined as persistent fever, unimproved or worsening clinical signs, and radiological findings after 72 h of standard therapy with macrolide antibiotics. All pediatric patients had indications for bronchoscopy and bronchoalveolar lavage.

### 2.2 Study methods

#### 2.2.1 Collection of clinical data

Clinical data were recorded for both groups of pediatric patients, primarily including: (1) General information: age and gender; (2) Clinical features: degree and duration of fever, length of hospital stay, duration of macrolide antibiotic use, frequency of bronchoalveolar lavage fluid (BALF) submissions for examination and lavage procedures, duration of post-admission steroid use, and extrapulmonary complications; (3) Laboratory tests: fasting peripheral venous blood was collected within 24 h of admission for laboratory indicators, including white blood cell count (WBC), percentage of lymphocyte count, platelet count (PLT), C-reactive protein (CRP), alanine aminotransferase (ALT), lactate dehydrogenase (LDH), procalcitonin (PCT), D-dimer, Mycoplasma load in BALF, and pathogen load in pharyngeal swabs.

#### 2.2.2 Bronchoscopy examination

All 73 pediatric patients diagnosed with MUMPP had indications for bronchoscopy and no contraindications for the procedure. In cases where bronchoalveolar lavage yielded insufficient therapeutic outcomes, endogenous foreign bodies were removed using biopsy forceps and cytology brushes. Concurrently, BALF specimens were collected for MP-DNA testing and pathogen culture.

### 2.3 Statistical methods

All data were statistically analyzed using the SPSS 26.0 software (SPSS Inc., Chicago, IL, United States). For data not following a normal distribution, intergroup comparisons were conducted using the Mann-Whitney U test. The findings from multifactorial logistic stepwise regression and lasso regression analysis were considered, along with the clinical significance of the indicators. Based on the findings of logistic regression analysis, a predictive model was constructed to forecast the probability of RMPP occurrence. Following multifactorial logistic regression analysis, the final predictive factors were utilized to construct a nomogram. The predictive performance was evaluated using the receiver operating characteristic (ROC) curve and decision curve analysis (DCA).

## 3 Results

### 3.1 Comparison of clinical signs and laboratory findings between the azithromycin group and the doxycycline group

A detailed comparison of clinical symptoms and laboratory parameters between the azithromycin group (*n* = 73) and the doxycycline group (*n* = 106) is presented in Table 1. The data are expressed as median (first quartile, third quartile). The doxycycline group demonstrated statistically significant clinical advantages in several key outcome measures. Specifically, patients treated with doxycycline experienced a shorter duration of fever after the initiation of study antibiotics [3.00 (1.00, 5.25) days vs. 4.00 (2.00, 7.00) days; *P* = 0.0468], required fewer bronchoalveolar lavage procedures [1.00 (1.00, 2.00) vs. 2.00 (1.00, 3.00); *P* = 0.0222], had a shorter overall hospital stay [10.00 (7.00, 13.25) days vs. 12.00 (9.00, 17.00) days; *P* = 0.0433], and required a shorter duration of systemic steroid therapy after admission [6.00 (5.00, 7.25) days vs. 7.00 (5.00, 10.00) days; *P* = 0.0434]. These four parameters, highlighted in bold in Table 1, were the only ones to show statistically significant differences between the two treatment groups. No statistically significant differences were observed between the two groups in baseline demographic characteristics, severity of illness at admission (as reflected by peak temperature, and most inflammatory and biochemical markers including CRP, PCT, LDH, and AST), or Mycoplasma pneumoniae load in either pharyngeal swabs or BALF (all *P* > 0.05). The Mann-Whitney U test was used for all intergroup comparisons of these non-normally distributed data (Table 1).

### 3.2 Univariate logistic regression analysis to identify factors associated with the occurrence of RMPP

This analysis was performed on data from the 106 pediatric patients diagnosed with RMPP (the study group) to identify factors associated with the development of refractory disease. Univariate Logistic regression analysis was conducted. The analysis revealed significant associations between RMPP and the following factors: the presence or absence of pleural effusion; levels of AST, fibrinogen degradation products, D-dimer, LDH, CRP, PCT, serum amyloid protein, ALT, and hemoglobin; the duration of macrolide antibiotic use outside the hospital; days of fever before hospital admission; peak temperature; and neutrophil percentage and lymphocyte percentage (all *P* < 0.05). All other factors did not demonstrate statistical significance (*P* > 0.05). In logistic regression, the regression coefficient (B) indicates the direction and magnitude of the association between a predictor and the log-odds of the outcome (RMPP). A positive coefficient suggests that as the predictor variable increases, the likelihood of RMPP increases, while a negative coefficient suggests a protective effect. The Wald statistic tests the significance of each coefficient. The odds ratio (OR), derived from the coefficient, quantifies the change in odds of the outcome for a one-unit increase in the predictor (for continuous variables) or for the presence of the factor (for categorical variables). An OR > 1 indicates increased risk, OR < 1 indicates decreased risk, and OR = 1 indicates no association (Table 2).

**TABLE 2:** Univariate Logistic regression analysis to identify factors associated with the occurrence of RMPP.

Variable	Coefficient	Standard error	Wald	Significance *P*-value	OR value (95%CI)
Presence of pleural effusion	2.124	0.436	**23.747**	**<0.001**	**8.37(3.56, 19.66)**
Gender	-0.517	0.382	**1.836**	0.175	**0.60(0.28, 1.26)**
Presence of fever	0.686	1.171	**0.343**	0.558	**1.99(0.20, 19.69)**
Serum mycoplasma antibody (0–20 Au/mL)	0.226	0.385	**0.345**	0.557	**1.25(0.59, 2.67)**
AST (15–40) U/L	0.010	0.004	**5.537**	**0.019**	**1.01(1.00, 1.02)**
SMEAN (fibrin degradation products μg/mL)	0.038	0.019	**4.103**	**0.043**	**1.04(1.00, 1.08)**
SMEAN (D-dimer (0–1) ug/mL)	0.077	0.038	**4.038**	**0.044**	**1.08(1.00, 1.16)**
Duration of macrolide antibiotic use outside hospital	0.207	0.058	**12.689**	**<0.001**	**1.23(1.10, 1.38)**
Days of fever before hospital admission	0.115	0.042	**7.577**	**0.006**	**1.12(1.03, 1.22)**
LDH (120–246 U/L)	0.002	0.001	**9.190**	**0.002**	**1.00(1.00, 1.00)**
CRP (0–8.2) ng/mL	0.117	0.024	**23.857**	**<0.001**	**1.12(1.07, 1.18)**
PCT ng/Ml	0.478	0.169	**8.000**	**0.005**	**1.61(1.16, 2.25)**
IL-6 (0–7 pg/mL)	0.000	0.001	**0.019**	0.889	**1.00(1.00, 1.00)**
SMEAN (serum amyloid A 0–10 mg/L)	0.004	0.001	**19.112**	**<0.001**	**1.00(1.00, 1.01)**
Age	–0.301	0.176	**2.920**	0.087	**0.74(0.52, 1.05)**
Peak temperature	1.024	0.340	**9.059**	**0.003**	**2.78(1.43, 5.42)**
Mycoplasma load in throat swab	0.000	0.000	**1.206**	0.272	**1.00(1.00, 1.00)**
Mycoplasma load in BALF	0.000	0.000	**2.447**	0.118	**1.00(1.00, 1.00)**
WBC 10^∧^9/L	0.095	0.053	**3.146**	0.076	**1.10(0.99, 1.22)**
RBC 10^∧^12/L	–0.560	0.330	**2.883**	0.090	**0.57(0.30, 1.09)**
Hemoglobin (g/L)	–0.053	0.020	**7.287**	**0.007**	**0.95(0.91, 0.99)**
Platelet count (10^∧^9/L)	–0.001	0.002	**0.235**	0.628	**1.00(1.00, 1.00)**
Neutrophil percentage (%)	0.076	0.019	**16.682**	**<0.001**	**1.08(1.04, 1.12)**
Lymphocyte percentage (%)	–0.085	0.022	**14.794**	**0.007**	**0.92(0.88, 0.96)**
Absolute neutrophil count (10^∧^9/L)	0.046	0.042	**1.201**	0.273	**1.05(0.96, 1.14)**
Absolute lymphocyte count (10^∧^9/L)	–0.328	0.175	**3.525**	0.060	**0.72(0.51, 1.01)**
ALT (9–50) U/L	0.009	0.004	**5.571**	**0.018**	**1.01(1.00, 1.02)**
Creatine Kinase (55–170) U/L	0.000	0.000	**1.555**	0.212	**1.00(1.00, 1.00)**
Creatine Kinase Isoenzyme Mass (0–5) ng/mL	–0.017	0.033	**0.271**	0.603	**0.98(0.92, 1.05)**

*P* < 0.05 indicates statistical significance, while *P* > 0.05 indicates no statistical significance. The Coefficient (B) represents the change in the log-odds of RMPP per unit change in the predictor. A positive B indicates a factor associated with higher risk of RMPP, a negative B indicates a protective factor. The Wald statistic tests the null hypothesis that *B* = 0. The Odds Ratio (OR) is calculated as exp(B); OR > 1 indicates increased odds of RMPP, OR < 1 indicates decreased odds. SMEAN refers to the standardized mean value of the variable, calculated by subtracting the mean and dividing by the standard deviation. This standardization was performed to facilitate the comparison of effect sizes across variables measured in different units in the regression model. Bold values indicate statistical significance (*P* < 0.05) in the univariate logistic regression analysis.

### 3.3 Development and validation of a predictive model for RMPP

To identify potential predictive factors for the subsequent Lasso regression, we first considered all clinical and laboratory variables collected (as listed in Table 1) that were biologically plausible or previously reported to be associated with severe or refractory MPP. The initial selection for inclusion in the Lasso regression model was not based on the statistical significance of differences between the doxycycline and azithromycin treatment groups (Table 1). Instead, the goal was to screen a broad set of candidate predictors for their individual association with the outcome (RMPP vs. non-RMPP) within the modeling subset. Clinical data, symptoms, and laboratory results from 44 pediatric patients diagnosed with RMPP were incorporated into a Lasso regression model to identify potential predictive factors for the development of RMPP among patients with macrolide-unresponsive disease. The Lasso regression applies a penalty that shrinks the coefficients of less important variables toward zero, effectively performing variable selection. As shown in Table 3, variables with non-zero coefficients were retained as potential predictors. Variables with non-zero coefficients were selected for inclusion in the predictive model. Specifically, the variables with non-zero coefficients identified by Lasso regression were: Presence of Pleural Effusion (coefficient = 0.394), Duration of Macrolide Antibiotic Use Outside Hospital (coefficient = 0.104), Days of Fever Before Hospital Admission (coefficient = 0.028), Lactate Dehydrogenase, or LDH (coefficient = 0.00015), CRP (coefficient = 0.034), PCT (coefficient = 0.017), and Serum Amyloid A (coefficient = 0.00058). The positive coefficients indicate that higher values of these variables are associated with increased risk of RMPP. Variables with a coefficient of zero (e.g., Gender, AST) were excluded from the subsequent model. From this Lasso-derived list of seven non-zero coefficient variables (Presence of Pleural Effusion, Duration of Macrolide Antibiotic Use Outside Hospital, Days of Fever Before Hospital Admission, LDH, CRP, PCT, and Serum Amyloid A), all were entered into a multivariable logistic regression model with a stepwise selection procedure (likelihood ratio). This subsequent step was crucial to identify a parsimonious set of independent predictors by testing the statistical significance of each variable while adjusting for others. The stepwise regression retained only four variables that maintained independent statistical significance (*P* < 0.05): the duration of macrolide antibiotic use outside the hospital, days of fever before hospital admission, CRP levels, and PCT levels. Variables such as Pleural Effusion, LDH, and Serum Amyloid A did not retain significant independent associations in the multivariable model and were therefore excluded to avoid overfitting and to enhance model simplicity and clinical utility. These four selected variables were then used to construct the final predictive model (Table 4). Ultimately, based on the clinical relevance of the variables, a logistic regression model was constructed to predict RMPP, incorporating indicators such as the presence of pleural effusion, AOPP, D-dimer, and the duration of the pre-hospital illness phase. The model’s chi-square value was validated using the Holm-Bonferroni method, yielding a value of 4.014 with a *P*-value of 0.856 (> 0.05), indicating significance. The model demonstrated excellent predictive capability, with an average C-value of 0.982 (95%CI: 0.981–0.984), suggesting robust predictive performance.

**TABLE 3:** Variable screening via Lasso regression analysis.

Variable	Coefficient
Intercept	–3.292166842
Presence of pleural effusion	0.394244385
Gender	0
Presence of fever	0
Serum mycoplasma antibody	0
AST	0
Fibrin degradation products	0
D-dimer	0
Duration of macrolide antibiotic use outside hospital	0.103765881
Days of fever before hospital admission	0.027635093
LDH	0.000149569
CRP	0.034144765
PCTngml	0.016976484
IL6	0
Serum amyloid A	0.000584787

**TABLE 4:** Multivariable logistic regression analysis of risk factors for RMPP.

Variable	*B*	Standard error	Wald	Significance	OR value (95%CI)
Duration of macrolide antibiotic use outside hospital	0.417	0.148	7.902	0.005	1.518(1.135, 2.031)
Days of fever before hospital admission	0.225	0.086	6.928	0.008	1.253(1.059, 1.481)
CRP (0–8.2) ng/mL	0.159	0.038	17.175	<0.001	1.172(1.087, 1.264)
PCT	0.417	0.214	3.786	0.052	1.518(0.997, 2.31)
Constant	–11.687	2.75	18.065	<0.001	

**FIGURE 1:**
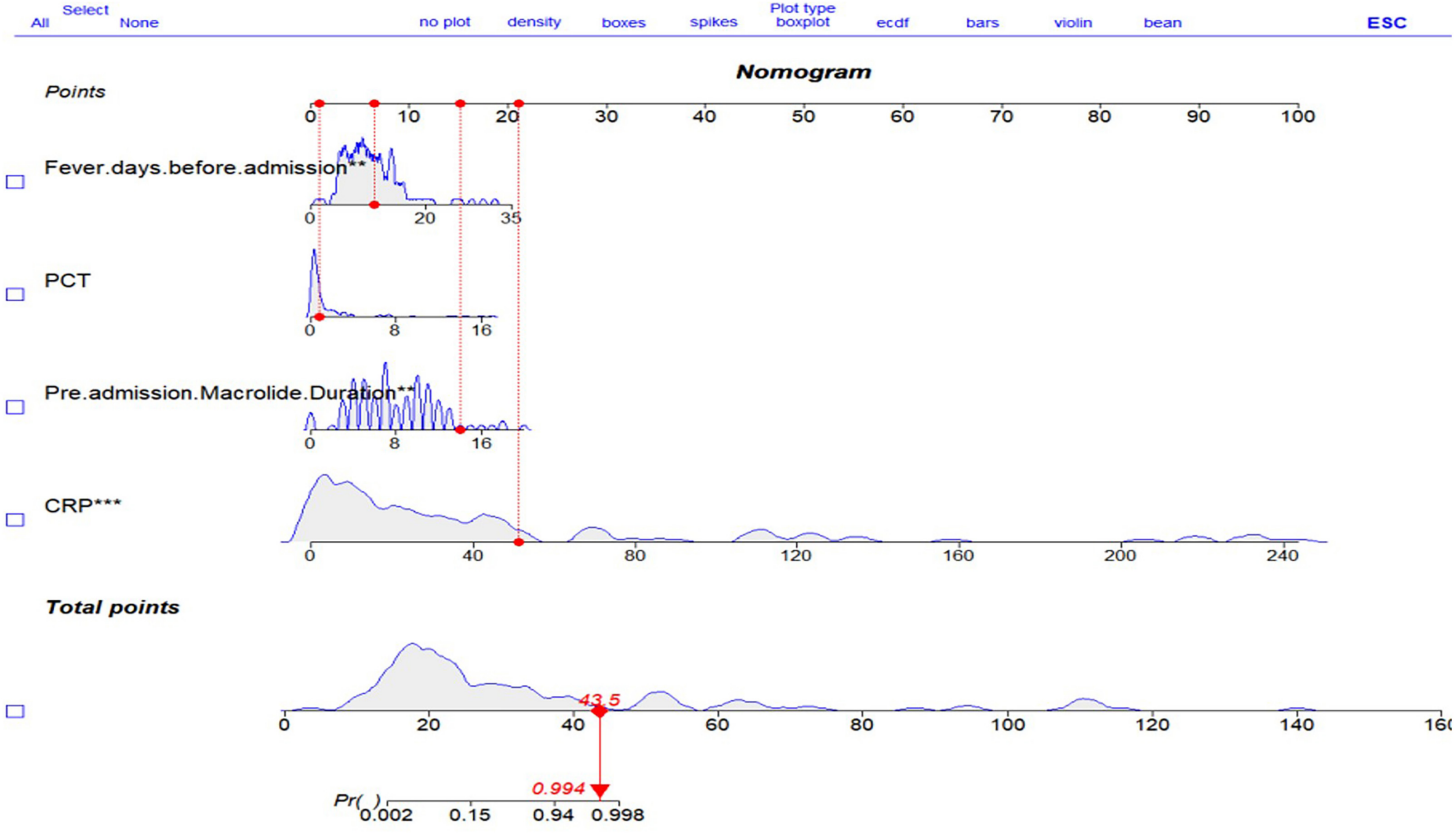
Nomogram incorporating duration of macrolide antibiotic use outside hospital, days of fever before hospital admission, PCT, and CRP.

The final multivariable logistic regression model was validated. The Holm-Bonferroni adjusted chi-square value was 4.014 (*P* = 0.856). The model demonstrated excellent predictive discrimination with a mean C-index (AUC) of 0.983 (95% CI: 0.981) of 0.983 nomogram was constructed based on this model (Figure 1). *P* < 0.05 indicates statistical significance, while *P* > 0.05 indicates no statistical significance.

### 3.4 Construction and validation of the nomogram model for predicting RMPP

**FIGURE 2:**
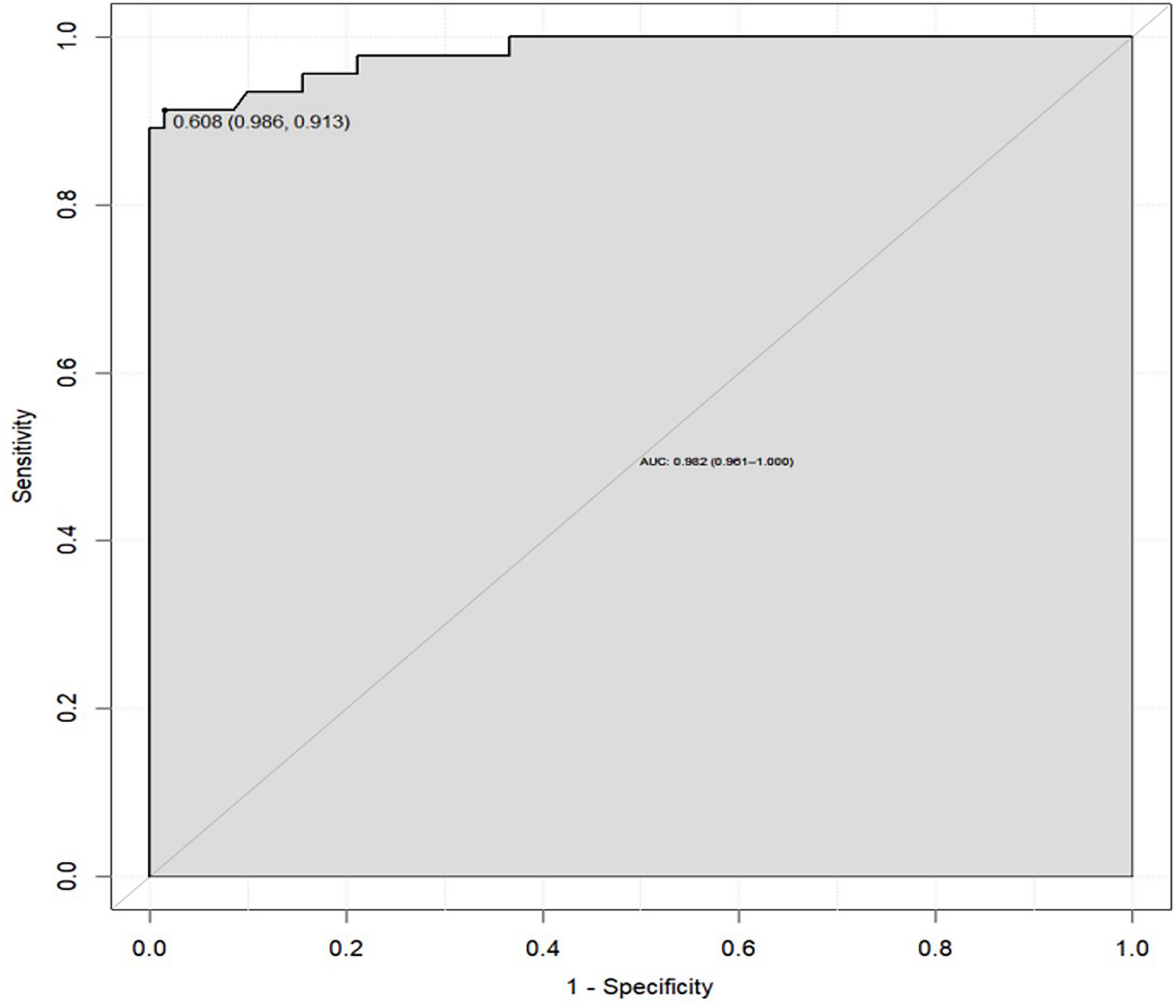
ROC curve of the nomogram for predicting RMPP (AUC = 0.906, 95% CI: 0.866–0.946).

**FIGURE 3:**
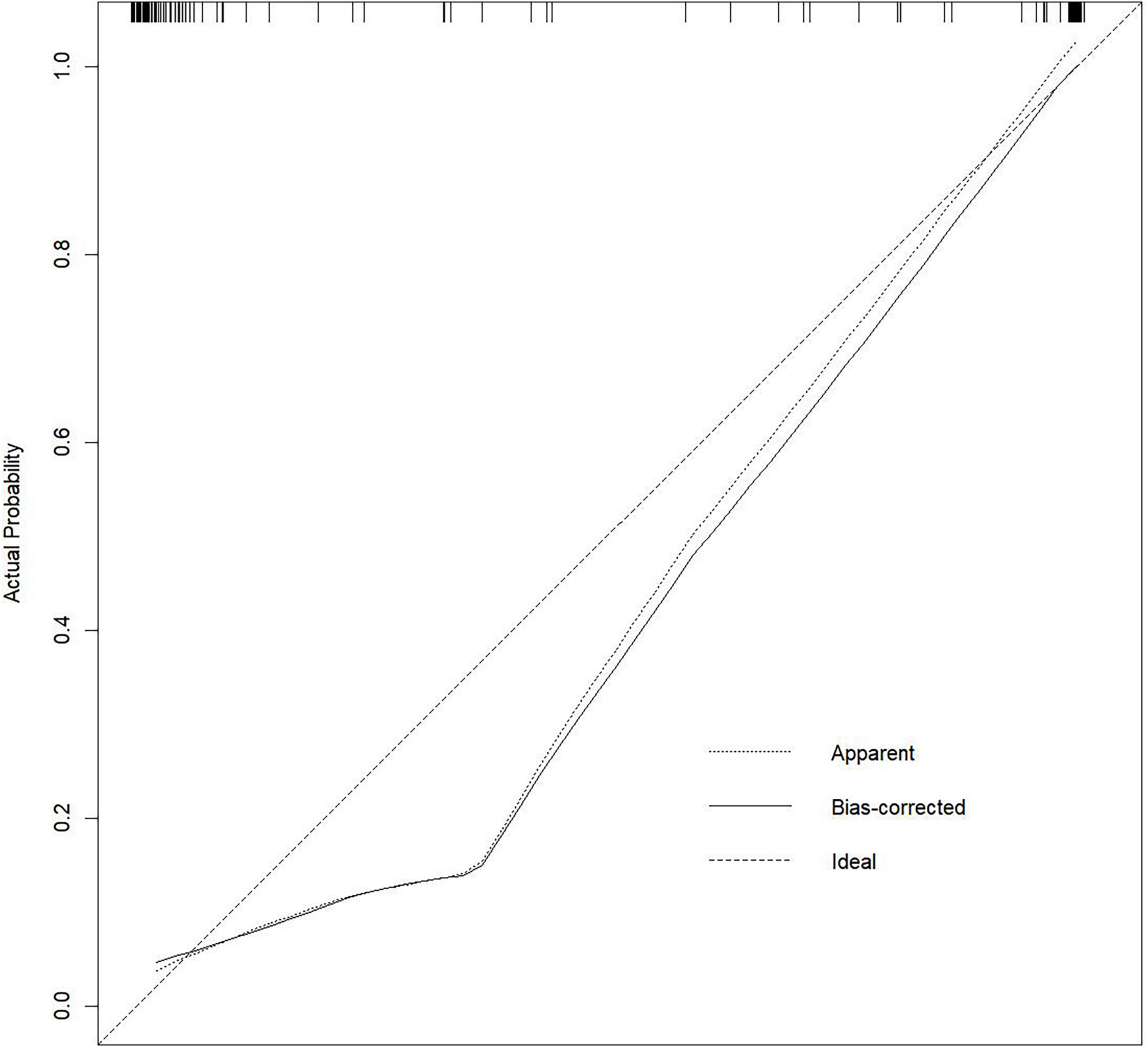
Calibration curve demonstrating good agreement between actual diagnoses of RMPP and predicted probabilities.

**FIGURE 4:**
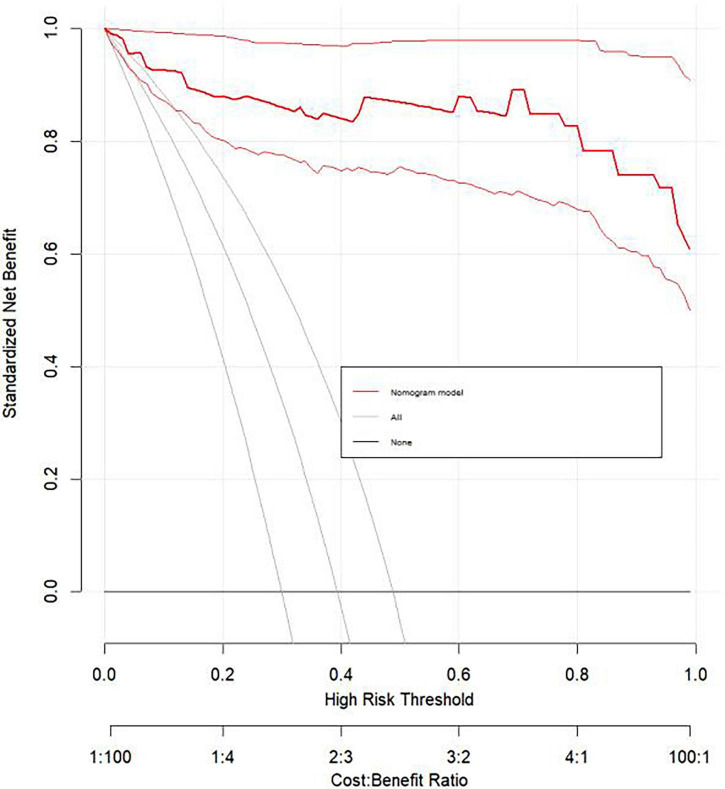
DCA of the predictive model.

Based on the LAOSS regression model and relevant literature, data from variables that were statistically significant and had a substantial impact on outcomes were further analyzed using logistic regression. Variables selected for this analysis included the duration of macrolide antibiotic use outside the hospital, number of days with fever before hospital admission, PCT, and CRP, which were used to construct the nomogram (Figure 1). Each predictor for each patient was assigned a score, distributed across the nomogram, with higher total scores indicating a greater likelihood of the child progressing to RMPP. The area under the ROC curve (AUC) for the nomogram was 0.982 (95% CI: 0.961–1.000), demonstrating excellent discriminative ability (Figure 2). The calibration curve, which approached the 45-degree line, showed commendable nomogram calibration (Figure 3). Additionally, the decision curve analysis (DCA) also indicated a positive clinical utility of this model (Figure 4).

## 4 Discussion

The incidence of macrolide resistance in MP has increased over time and is associated with the occurrence of refractory Mycoplasma pneumoniae pneumonia (RMPP). Recent guidelines propose alternative treatment options for RMPP, primarily second-line antibiotics such as tetracyclines (e.g., doxycycline or minocycline) and fluoroquinolones (1). However, the effective management of macrolide-resistant pediatric MP pneumonia remains challenging due to limitations and concerns associated with these alternative agents in children. This retrospective study included a total of 179 pediatric patients with macrolide-unresponsive Mycoplasma pneumoniae pneumonia (MUMPP). The comparative analysis of clinical outcomes (e.g., fever duration, hospital stay) between second-line and continued macrolide therapy was performed on all 179 patients, comprising 106 patients treated with doxycycline (RMPP group) and 73 patients treated with azithromycin (MUMPP group). For the specific purpose of developing and validating a predictive model for RMPP risk, a focused modeling subset was utilized, which included data from 44 patients selected from the RMPP group along with the data from the azithromycin-treated group (as non-RMPP controls). The outcomes demonstrated that in MUMPP cases, the doxycycline group showed statistically significant improvements in terms of shorter fever duration, fewer lavage procedures, reduced steroid use, and decreased length of hospital stays compared to the azithromycin group. These findings are consistent with those of Lee et al. (6), who reported that doxycycline demonstrated superior efficacy over macrolides in treating pediatric patients with RMPP, reducing fever duration and accelerating chest radiograph improvement. Okada et al. (7) also found doxycycline superior in 24-h fever resolution and MP culture positivity rates after 5 days of therapy compared to minocycline and macrolides. The superior clinical performance of doxycycline in this context can be attributed primarily to the markedly lower prevalence of Mycoplasma pneumoniae resistance to tetracyclines compared to macrolides. Macrolide resistance in MP, mediated mainly by point mutations in the 23S rRNA gene, has become highly prevalent due to their decades-long use as first-line therapy for pediatric respiratory infections (5). In contrast, tetracyclines like doxycycline have not been used as first-line agents for MP in children historically, owing to concerns about dental discoloration, resulting in minimal selective pressure and consequently very low reported resistance rates worldwide (6). The distinct mechanism of action (binding to the 30S ribosomal subunit) and the rarity of acquired resistance genes (e.g., tetM) in clinical MP isolates further support the sustained susceptibility to this class. Additionally, doxycycline’s favorable pharmacokinetic properties, including good tissue penetration into the lungs, and its documented anti-inflammatory effects may synergistically contribute to a more effective and rapid clinical response in macrolide-resistant infections. Nonetheless, Xie et al. (8) noted that for pediatric patients with MUMPP who are predicted to have a lower risk, continuation of macrolide therapy, rather than switching to second-line antibiotics such as tetracyclines (e.g., doxycycline or minocycline) or fluoroquinolones, should still be considered. Existing studies have recognized the efficacy and safety of doxycycline in pediatric patients, particularly for moderate to severe cases, recommending the use of macrolides or tetracyclines (suitable for pediatric patients over 7 years old) (9–11). Despite macrolides being the first-line treatment for pediatric MP pneumonia, the differences in hospital stay duration, number of lavage procedures, steroid usage, and hospitalization days support the timely transition to doxycycline during treatment.

Regarding the clinical characteristics of pediatric patients with MUMPP and RMPP, we noted that age, gender, thoracic manifestations, and the need for oxygen therapy were not influenced by macrolide resistance. Additionally, laboratory findings, including elevated serum amyloid A, PCT, and CRP, showed no significant differences, aligning with the data reported in the literature (11–15). Therefore, distinguishing between MUMPP and RMPP based on clinical features and laboratory data has its limitations.

Due to the potential for permanent dental discoloration, the use of tetracyclines in pediatric patients under the age of eight is generally restricted. However, recent studies suggest that doxycycline is less likely to cause visible tooth staining or enamel hypoplasia in young pediatric patients (16). On this basis, the American Academy of Pediatrics indicates that doxycycline can be used to treat MP pneumonia in patients of any age, as it may help to reduce the duration of hospital stays. Nevertheless, caution is advised due to the photosensitivity reactions associated with doxycycline, and excessive exposure to sunlight should be avoided (17). Although doxycycline is not recommended as the first-line treatment for pediatric MP pneumonia, our findings, together with existing literature, support considering its early use as an effective second-line agent in pediatric patients with confirmed or highly suspected macrolide-resistant RMPP. This decision requires careful clinical judgment, weighing the proven benefits in overcoming resistance and improving clinical outcomes, as shown in this study, against the known potential risks associated with tetracyclines (e.g., dental discoloration, photosensitivity). In the context of worsening disease due to macrolide unresponsiveness, the risks of delayed effective therapy may outweigh the concerns regarding short-course doxycycline use in children over 8 years of age, for whom recent guidelines have acknowledged its acceptable safety profile (17). Nevertheless, the decision to switch to doxycycline should be individualized.

In this report, we employed univariate Logistic regression analysis to identify RMPP in pediatric patients with MUMPP who continued to receive macrolide therapy post-confirmation of MUMPP. The findings indicated that the following factors were significantly associated with the occurrence of RMPP: the presence of pleural effusion, AST levels, fibrinogen degradation products, D-dimer levels, duration of macrolide antibiotic use outside the hospital, days of fever before hospital admission, LDH levels, CRP levels, PCT levels, serum amyloid protein levels, peak temperature, and ALT levels (*P* < 0.05). This is consistent with the findings reported by Liu et al. (18), where fever duration, CRP, LDH, D-dimer, ESR, large patchy shadows, and pleural effusion were identified as independent risk factors for RMPP. CRP, an acute-phase reactant protein, increases in response to intense immune-inflammatory reactions and can be used to assess the severity of the condition (19). It is believed that a CRP level > 40 mg/L should prompt consideration of potential progression in MPP (18). LDH, a glycolytic enzyme, is released extracellularly when cells lyse or cell membranes are compromised, leading to elevated serum LDH levels (18). Research by Lee et al. (20) has shown that serum LDH is a reliable indicator of the severity of lung damage. Furthermore, it has been reported (21) that higher procalcitonin levels are associated with more severe infections. Procalcitonin levels can serve as a reference for antibiotic therapy in pediatric patients with *Mycoplasma pneumoniae*, providing guidance for medication use. Therefore, for pediatric patients with MPP who exhibit persistent fever, concurrent pleural effusion and extrapulmonary complications, significant elevation in various inflammatory markers, prolonged disease course, and poor response to macrolide therapy, clinicians should be highly vigilant for the occurrence of RMPP.

It is important to note that the risk factors identified in this study were derived from a pediatric population that was otherwise generally healthy prior to MP infection, as per our exclusion criteria which ruled out significant underlying immunocompromising conditions, autoimmune, hematologic, or other systemic severe diseases. This strengthens the inference that the elevated inflammatory markers (e.g., CRP, PCT) and other associated factors are more likely to be direct correlates of the excessive host inflammatory response and tissue damage in RMPP itself, rather than being attributable to confounding baseline comorbidities.

Our study utilized univariate and multivariate Logistic regression analyses to identify risk factors for Refractory *Mycoplasma pneumoniae* Pneumonia (RMPP) among patients with *Mycoplasma pneumoniae* Pneumonia (MPP). Based on these risk factors, a nomogram was constructed, and its predictive performance was evaluated using Receiver Operating Characteristic (ROC) curves and Decision Curve Analysis (DCA) (22). Our findings indicate that the duration of macrolide antibiotic use outside the hospital, days of fever before hospital admission, Procalcitonin (PCT), and C-reactive protein (CRP) are critical risk factors included in the nomogram. Cheng et al. (23) identified lactate dehydrogenase (LDH), albumin, and high fever as independent predictors of RMPP risk in pediatric patients with MPP. Another study demonstrated that age, combined with days of fever, CRP, glutamic oxaloacetic transaminase (GOT), LDH, and chest imaging scores, could effectively identify RMPP at an early stage (24). This tool can assist in the early estimation of RMPP risk in pediatric MPP patients, enabling the initiation of effective treatment sooner, potentially improving the clinical outcomes of RMPP.

It is important to contextualize our findings within the global epidemiology of Mycoplasma pneumoniae. The high prevalence of macrolide-resistant MP (MRMP) in Asia, particularly in East Asia, which formed the setting for this study, contrasts with the notably lower rates reported in many Western countries (e.g., often below 10% in Europe and North America) (25, 26). This epidemiological disparity directly impacts clinical management strategies. In regions with low MRMP prevalence, macrolides remain highly effective first-line agents, and the threshold for switching to second-line antibiotics like doxycycline is appropriately higher. Consequently, the predictive model we developed, which incorporates factors like prolonged pre-admission macrolide use and fever, is most immediately relevant for clinical settings in high-prevalence areas facing frequent macrolide-unresponsive cases. However, the fundamental pathophysiology it aims to capture—an excessive or dysregulated host inflammatory response leading to refractory disease—is a universal concern in MP pneumonia. The specific biomarkers (e.g., CRP, PCT) and clinical timelines identified may serve as a reference, but their optimal cut-off values and relative weights might require adjustment or validation in populations with different baseline resistance rates and circulating strains.

Despite the advantages highlighted for doxycycline in RMPP therapy and the construction of an effective RMPP predictive model, this study still has several limitations. Firstly, as a retrospective analysis, there is an inherent risk of selection and information biases, and the relatively small sample size may affect the generalizability of the results. Secondly, although we have identified several significant risk factors, there is still the possibility that some potential key factors were omitted. Therefore, future studies should involve multicenter, large-scale prospective research to validate the conclusions of this study and further refine the predictive model. Moreover, while comparisons of different treatment regimens within this study highlighted the advantages of doxycycline, ethical and clinical practice constraints precluded the execution of a randomized controlled trial. Consequently, while doxycycline shows promising therapeutic effects, further research is needed to support its long-term safety and resistance profiles in pediatric patients.

## 5 Conclusion

This study evaluated the efficacy of doxycycline in the treatment of pediatric RMPP and compared it with azithromycin. The results demonstrated that, for cases resistant to MP, doxycycline was associated with significantly reduced duration of fever, fewer lavage procedures, shorter steroid use, and shorter hospital stay, suggesting superior clinical efficacy in this specific, resistant population. Furthermore, through regression analysis and the Lasso regression model, key risk factors associated with the occurrence of RMPP were identified, and an effective predictive model was established, showing high predictive capability (AUC = 0.982). This study provides supporting evidence that doxycycline can be an effective therapeutic alternative for pediatric RMPP when macrolide resistance is suspected or confirmed. However, its use must be balanced against known safety considerations. Future research, particularly prospective randomized controlled trials, is needed to definitively establish the efficacy, optimal timing, and long-term safety profile of doxycycline in this population, and to further validate the clinical utility of the predictive model for personalized treatment decisions.

## Data Availability

The original contributions presented in the study are included in the article/supplementary material, further inquiries can be directed to the corresponding author.
